# Modeling and Targeting Alzheimer’s Disease With Organoids

**DOI:** 10.3389/fphar.2020.00396

**Published:** 2020-03-31

**Authors:** Angelos Papaspyropoulos, Magdalini Tsolaki, Nicolas Foroglou, Anastasia A. Pantazaki

**Affiliations:** ^1^ Laboratory of Biochemistry, Department of Chemistry, Aristotle University of Thessaloniki, Thessaloniki, Greece; ^2^ 1st Department of Neurology, AHEPA University Hospital, Thessaloniki, Greece; ^3^ Department of Neurosurgery, AHEPA University Hospital, Aristotle University of Thessaloniki, Thessaloniki, Greece

**Keywords:** Alzheimer’s disease, disease modelling, hPSC-derived brain organoids, pharmacological treatments, primary tissue-derived organoids

## Abstract

Human neurodegenerative diseases, such as Alzheimer’s disease (AD), are not easily modeled *in vitro* due to the inaccessibility of brain tissue and the level of complexity required by existing cell culture systems. Three-dimensional (3D) brain organoid systems generated from human pluripotent stem cells (hPSCs) have demonstrated considerable potential in recapitulating key features of AD pathophysiology, such as amyloid plaque- and neurofibrillary tangle-like structures. A number of AD brain organoid models have also been used as platforms to assess the efficacy of pharmacological agents in disease progression. However, despite the fact that stem cell-derived brain organoids mimic early aspects of brain development, they fail to model complex cell-cell interactions pertaining to different regions of the human brain and aspects of natural processes such as cell differentiation and aging. Here, we review current advances and limitations accompanying several hPSC-derived organoid methodologies, as well as recent attempts to utilize them as therapeutic platforms. We additionally discuss comparative benefits and disadvantages of the various hPSC-derived organoid generation protocols and differentiation strategies. Lastly, we provide a comparison of hPSC-derived organoids to primary tissue-derived organoids, examining the future potential and advantages of both systems in modeling neurodegenerative disorders, especially AD.

## Introduction

Alzheimer’s disease (AD) constitutes the most prominent cause of late-life dementia, affecting over 50 million individuals. Additionally, AD represents one of the leading causes of death worldwide (Collaborators, G.B.D.D, 2019). Although considerable progress has been made in neuroscience, there are currently no available drug treatments curing the disease, thus highlighting that it is accompanied by significant social and economic burden (Vigo et al., 2016; Amin and Pasca, 2018). The majority of AD clinical cases develop symptoms beyond the age of 65 and are collectively referred to as sporadic AD (SAD). Familial AD (FAD) incidents, which pertain only to 2–5% of AD cases, develop early-onset symptoms and have been linked to mutations in genes such as *APP, PSEN1,* and *PSEN2* (Holtzman et al., 2011).

AD is caused by neuronal deposition and subsequent toxicity of amyloid-beta (Aβ)- and tau hyperphosphorylation-derived neurofibrillary tangles (NFTs) (Palmer, 2011; Dos Santos Picanco et al., 2018; Yan et al., 2019). In the AD brain, Aβ plaques are formed by aggregation of monomeric Aβ peptides into toxic Aβ oligomers, which subsequently generate the insoluble fibrils. Aβ plaque formation has been shown to trigger inflammatory responses and Reactive Oxygen Species (ROS) production, resulting in neuronal death (Prokop et al., 2013; Heppner et al., 2015; Yan et al., 2019). Additionally, toxic Aβ species may trigger caspase-associated apoptosis, following their transfer into neuronal cells (Prokop et al., 2013; Heppner et al., 2015; Yan et al., 2019). In healthy individuals, β- and γ-secretases proteolyze the amyloid precursor protein (APP) to soluble and non-toxic Aβ monomers, whereas in AD patients, Aβ plaques are formed due to increased production or insufficient removal of Aβ peptides (Bekris et al., 2010). Moreover, extracellular matrix (ECM) components such as heparin sulfate proteoglycans (HSPG) have been shown to foster amyloid plaque formation (van Horssen et al., 2002). Aβ peptide accumulation may synergize with tau-related NFT formation to contribute to AD manifestation, as indicated by a number of studies (Nisbet et al., 2015).

Several limitations accompany the implementation of transgenic mice in elucidating the molecular mechanisms underlying AD pathophysiology, such as the inability to capture tau pathology and the development of AD features early in life (Andorfer et al., 2003; Kitazawa et al., 2012; Sasaguri et al., 2017; Gerakis and Hetz, 2019). Additionally, monolayer neuronal cultures from AD patients lack plaques and tangles and express toxic proteins, which also limit their potential use as model systems (Amin and Pasca, 2018). Thus, novel systems are required to model AD development and serve as platforms for the discovery of effective AD treatments. In this literature review, we aim to provide an overview of recent advances regarding the development of brain organoids as a humanized model system against AD.

### iPSCs in AD Modeling

The establishment and optimization of protocols allowing the reprogramming of human somatic cells into induced pluripotent stem cells (iPSC) opened new avenues in disease modeling (Tiscornia et al., 2011). Human pluripotent stem cells (hPSC) include blastocyst-derived human embryonic stem cells (ESC) and hiPSCs reprogrammed from somatic cells. HPSCs display unlimited self-renewal and can differentiate toward mesoderm, endoderm, or ectoderm (Rowe and Daley, 2019). Three methods have been so far established to capture the AD phenotype using hPSCs. The first method pertains to chemical induction with Aβ42 oligomers or Aβ42 inducers, such as aftin5. In this method, neural cells derived from AD-free hPSCs are induced to develop AD phenotypes (Vazin et al., 2014; Pavoni et al., 2018). Although certain pathophysiological features of the disease such as neuronal cytotoxicity can be displayed by implementing this method, induced neuronal cells usually lack other features such as extracellular Aβ plaque formation. The second method is based on the generation of iPSCs from somatic cells carrying known AD mutations and subsequent differentiation of those iPSCs into various types of neuronal cells. iPSCs deriving from FAD patients usually carry *PS1, PS2, or APP* genomic mutations, whereas those deriving from SAD patients carry *APOE4* mutations (Muratore et al., 2014). In the third method, lentiviral transduction or CRISPR-Cas9-mediated genomic editing are implemented in order to induce overexpression or expression of mutant APP, PS1, PS2, and APOE4 proteins in healthy hPSCs (Koch et al., 2012; Huang et al., 2017). Additionally, by utilizing human ESC-derived neurons ectopically expressing APOE2/E3/E4, it was shown that all APOE isoforms could induce Aβ and APP production, albeit to a different extent, with APOE4 being the most potent isoform (Huang et al., 2017). The majority of hPSC-based AD models implemented either two-dimensional (2D) or embryoid body (EB) differentiation methodologies to produce different types of neurons, including forebrain, cortical glutamatergic, GABAergic, and cholinergic neurons (Harasta and Ittner, 2017; Jorfi et al., 2018).

2D cell culture models of FAD and SAD based on patient-derived iPSCs have been shown to resemble some features of AD pathophysiology, such as intracellular accumulation of soluble Aβ species, aggregation of insoluble Aβ species, and tau hyperphosphorylation (Kondo et al., 2013; Freude et al., 2014). Moreover, iPSC-derived neurons from FAD patients can successfully capture important features of AD pathogenesis at early stages (Israel et al., 2012). However, while iPSC- or ESC-derived neurons cultured in monolayer have yielded important findings, they fail to present various morphological and functional properties of the human brain, which poses limitations in their use as model systems for neurodegenerative diseases. Neuronal maturation and development of synapse connections are governed by cell-cell and ligand-receptor signaling, which are not sufficiently established when neurons are cultured in monolayer (Amin and Pasca, 2018). Monolayer cultures do not offer accurate representations of the number, functional interactions, and regulatory functions typically observed in oligodendrocytes, astrocytes, and microglia in the human brain. Additionally, it is difficult to faithfully mimic neuronal maturation in monolayer cultures, as the *in vivo* process takes place on much longer timescales than monolayer cultures can be maintained (Dehaene-Lambertz and Spelke, 2015; Silbereis et al., 2016). In the case of AD, in particular, 2D cultures fail to display aggregation of extracellular β-amyloids, as only low Aβ species levels are produced even in the presence of the most prominent FAD genetic mutations. Moreover, the absence of interstitial compartment is believed to inhibit extracellular β-amyloid aggregation in 2D cultures (Choi et al., 2014).

### Modeling AD With hPSC-Derived Organoids

**Figure 1:**
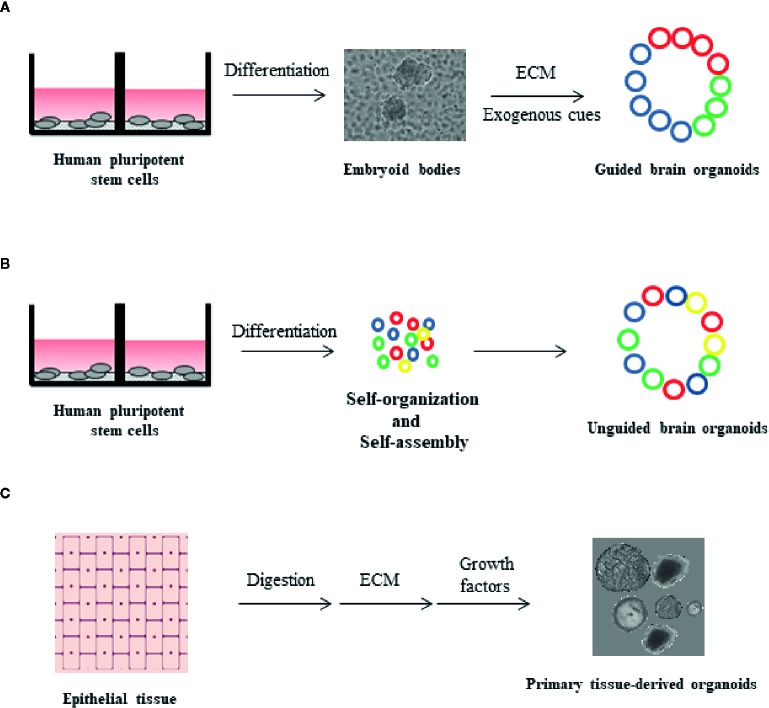
Organoid formation technologies from human pluripotent stem cells (hPSCs) and primary tissue. **(A)** Guided brain organoids generated from hPSCs through embryoid body (EB) formation. The process requires extrinsic factors, such as extracellular matrix (ECM) and exogenous differentiation signals. The presence of different cell types in the organoids is indicated by different colors (blue, red and green) **(B)** Unguided brain organoids generated from hPSCs upon stem cell self-organization and self-assembly, in the absence of extrinsic factors. With this method, non-ectodermal cell types may be incorporated in brain organoids (yellow color) **(C)** Primary tissue-derived organoids are generated by human epithelial tissue of any age. Described protocols include tissue digestion and subsequent use of defined cell culture media supplemented with tissue-specific growth factors. Organoids are embedded in ECM.

The limitations of monolayer cultures triggered the development of additional *in vitro* model systems capable of resembling human brain architecture and function more accurately than before (Nakano et al., 2012; Muguruma et al., 2015). The improvement of protocols for *in vitro* iPSC differentiation led to the establishment of “organoids”, which are three-dimensional (3D) self-organized structures displaying morphological and functional similarities with complex organs, such as the brain. Brain organoid formation relies on the self-organization ability of hiPSCs, which may be facilitated by additional exogenous components, for example matrigel (Mansour et al., 2018; Pham et al., 2018). Brain organoids develop to display organized structures, resembling distinct regions of the brain, thus maintaining hallmarks of key developmental processes involved in brain formation (Lancaster et al., 2013). Over the past few years, various attempts have been made to model specific brain substructures with the use of organoids. In this context, forebrain, midbrain, hippocampus, and retinal organoids have been developed from hiPSCs (Di Lullo and Kriegstein, 2017). A major point of discussion regarding the optimization of organoid formation protocols is whether cell fate induction should be facilitated through the addition of exogenous morphogens and signaling molecules or not facilitated at all. Several protocols favor spontaneous neural induction by avoiding supplementation of organoid media with exogenous factors, thereby resulting in the acquisition of heterogeneous cell populations, corresponding to various brain regions (Lancaster et al., 2013; Camp et al., 2015; Quadrato et al., 2017). Undirected organoids, often grown in ECM, stochastically give rise to cells corresponding to multiple brain sections ranging from the retina to hindbrain (**Figure 1A**) (Lancaster et al., 2013). One major limitation of spontaneous neural induction is that a proportion of cells are randomly differentiated into non-ectodermal cell types (Camp et al., 2015; Quadrato et al., 2017). Hence, most current efforts are based on protocols optimizing the application of extrinsic cues to induce neuronal differentiation.

In guided brain formation, defined combinations of exogenously applied factors direct the *in vitro* specification of stem cell aggregates into organoids (**Figure 1B**) (Pasca et al., 2011; Mariani et al., 2015; Qian et al., 2016; Amin and Pasca, 2018). Guided methodologies for brain organoid generation were first described by the Sasai group, which conceived and optimized targeted 3D differentiation protocols based on culturing EB aggregates in serum-free conditions (Eiraku et al., 2008; Danjo et al., 2011; Muguruma et al., 2015; Sakaguchi et al., 2015). Directed organoid cultures have the advantage of containing different cell lineages at relatively stable proportions, thereby limiting potential variations across different batches and cell lines (Sloan et al., 2017). Organoids mature over a period of many months (Sloan et al., 2017), achieving a diameter of several millimeters, and contain heterogeneous cell types including neuronal subtypes, outer radial glia cells, astrocytes, and oligodendrocytes (Camp et al., 2015; Qian et al., 2016; Birey et al., 2017; Quadrato et al., 2017; Sloan et al., 2017; Amin and Pasca, 2018).

In order to model inter-regional interactions pertaining to brain physiology, several groups have attempted to differentiate hPSCs toward distinct brain region-specific organoids before fusing them together to allow the formation of “assembloids” integrating multiple region identities (Bagley et al., 2017; Birey et al., 2017; Xiang et al., 2017). Along those lines, assembloids have formed *via* fusion of dorsal and ventral forebrain organoids (Birey et al., 2017). In those structures, intraneurons originating from the ventral region translocate to the dorsal region, thus resembling the *in vivo* situation.

Brain organoids generated from hPSCs have been recently implemented to model various neurological disorders such as autism (Mariani et al., 2015; Birey et al., 2017), microcephaly (Tiscornia et al., 2011), Parkinson’s disease (Kim et al., 2019), and Zika virus infections (Qian et al., 2016). The first successful attempt in using organoids to model AD was based on human neuronal progenitor cells genetically manipulated to overexpress mutant PS1 and APP (Choi et al., 2014). This methodology allowed the simultaneous presence of β-amyloid- and tau-related features in a single 3D model system. Those 3D structures carrying FAD mutations displayed increased detergent-resistant accumulations of phosphorylated tau, together with filamentous tau.

A sophisticated model of AD cerebral organoids was recently generated from FAD patient- or Down patient-derived iPSCs (Gonzalez et al., 2018). In this model, brain organoids displayed progressive accumulation of amyloidogenic Aβ peptides, accompanied by the development of structures strongly resembling amyloid plaques and NFTs. These phenotypes were absent in cerebral organoids derived from “control” templates such as healthy hiPSC, mouse ESCs, or mouse iPSCs (Gonzalez et al., 2018).

Recently a new 3D human tri-culture model including neurons, astrocytes, and microglia was developed to model AD with the use of microfluidics (Park et al., 2018). The model displayed critical features of AD pathology, such as β-amyloid aggregation, tau hyperphosphorylation, neuroinflammatory activity, microglial recruitment, axonal cleavage resulting from neurotoxic activities, and release of NO with deleterious effects on AD neurons and astrocytes (Park et al., 2018).

### Use of hPSC-Derived Organoids as a Treatment Platform for AD

Two studies have implemented AD brain organoids in order to assess the effect of pharmacological agents on various disease features. Both studies used primarily modulators of β- or γ-secretase and were able to observe reductions in Aβ peptide levels, as well as alterations in tau pathology, in line with previous reports involving iPSCs. Choi et al. (2014) developed 3D-differentiated neuronal cells carrying FAD mutations, and importantly, demonstrated that perturbation of β-amyloid generation with β- or γ-secretase inhibitors attenuated both β-amyloid and tau-related pathology, indicating that tau-dependent phenotypes may be driven by excessive accumulation of Aβ species as a result of FAD mutations (Choi et al., 2014). Additionally, the use of glycogen synthase kinase 3 (GSK3) was found to regulate β-amyloid-mediated tau phosphorylation in that system. Thus, that study constituted the first attempt to show that stem cell-derived 3D *in vitro* systems can potentially serve as drug treatment platforms against AD (Choi et al., 2014).

In a more recent study by Raja et al. (2016), hPSC-derived organoids from FAD patients again exhibited AD-like pathophysiological features, including amyloid aggregation, tau hyperphosphorylation, and endosome abnormalities, in an age-dependent fashion (Raja et al., 2016). The authors showed that the 3D system they developed could be easily subjected to experimental manipulation and serve as a potential drug treatment platform. The authors found that treatment of FAD patient-derived organoids with γ-secretase inhibitor compound E or BACE-1 β-secretase inhibitor (β-secretase inhibitor IV) partially reversed both amyloid and tau pathology. Additionally, in contrast to published data not supporting a pivotal role of amyloids in AD manifestation (Takahashi et al., 2015; Kametani and Hasegawa, 2018), the authors showed that inhibition of Aβ species limited tau hyperphosphorylation only after Aβ reduction was observed, suggesting that Aβ accumulation-driven phenotypes in AD may emerge prior to tauopathy (Raja et al., 2016).

### Limitations of hPSC-Derived Organoids in Modeling AD

HPSC-derived brain organoids display most of the advantages of 2D cultures, while offering the ability to model complex cell-cell interactions, as they usually contain more than one cell population. Because of their advantages, hPSC-derived brain organoids have been utilized to model AD and examine the impact of pharmacological factors on disease progression, however, serious technical hurdles are still required to be resolved. Additionally, the organoid generation technology applied so far to model neurodegenerative diseases, including AD, needs to be reviewed and updated.

One critical limitation to modeling AD with the use of hPSC-derived organoids relates to aging. Aging constitutes the main risk factor to develop AD, especially in the case of SAD, and the process of aging is accompanied by numerous genetic alterations resulting in changes in the overall cellular transcriptional profile (Lopez-Otin et al., 2013; Gerakis and Hetz, 2019). However, iPSC-derived neural cells display a transcriptional profile similar to prenatal brain (Camp et al., 2015; Gerakis and Hetz, 2019), thereby making it challenging to recapitulate aging-related phenotypes.

Another important limitation is the lack of complete vascularization. Vascularization is critical to mimic the *in vivo* situation in the brain, as maturation of neuronal cells cannot be accomplished without sufficient oxygen and nutrient supply. Insufficient neuronal cell maturation results in perturbed synapse formation, whereas lack of vascularization overall limits organoid culturing periods (Lancaster et al., 2013; Di Lullo and Kriegstein, 2017). Lack of vascularization additionally prevents modeling important aspects of brain physiology, such as the blood brain barrier (Huch et al., 2017). Along these lines, cerebral organoids produced from AD- or Down patient-derived iPSCs structurally resemble the human cortex, however they contain only neurons and glial cells, lacking oligodendrocytes. Additionally, those organoids fail to establish active synapses (Gonzalez et al., 2018). To overcome vascularization-related hurdles, heterotypic cultures combining mouse brain cells or brain progenitors with endothelial and mesenchymal stem cells have been recently used to generate 3D organ buds (Takebe et al., 2015), however, the functionality of that system has yet to be addressed in mice and humans. Additionally, it has been shown that although brain organoids are able to incorporate exogenous endothelial cells, the resulting endothelial network may not be functional (Pham et al., 2018).

HPSC-derived organoid models are so far challenged by low reproducibility and homogeneity. Organoid differentiation protocols relying on hPSC self-organization, in particular, lead to variable outcomes. Brain organoids differ from each other in size and structure, which are limiting factors in accurately modeling diseases such as AD. The small size, in particular, of hPSC-derived organoids comprises an important limitation in modeling human brain development, especially at later stages (Rambani et al., 2009). Microfluidics, spinning bioreactors and orbital shakers combining new biomaterials and culture methodologies, have been proposed as new avenues to control neural patterning more accurately and improve oxygen and nutrient supply to the organoid interior (Kadoshima et al., 2013; Qian et al., 2016; Lancaster et al., 2017; Yan et al., 2019).

Improvements in culturing conditions and the use of novel biomaterials might also help rectify another important limitation encountered in hPSC-derived organoid cultures, which is insufficient immune cell representation. Several brain organoid systems developed so far are characterized by the presence of astrocytes, but no microglial cells (Yakoub, 2019). The absence of microglial cells could be also attributed to their distinct embryonic origin, as they derive from yolk sac erythromyeloid precursors (Ginhoux and Prinz, 2015; Li and Barres, 2018).

HPSC-derived organoids predominantly rely on the process of somatic cell reprogramming, which has been extensively linked to increased risk of genomic instability, as iPSCs may often carry mutations related to known tumorigenic loci (Mayshar et al., 2010; Hussein et al., 2011; Laurent et al., 2011). This implication poses serious limitations to the use of hPSC-derived organoids in modeling human disease. Additionally, genomic analyses of early passage iPSCs have indicated that they might retain “epigenetic memory” related to their previous fate, by displaying DNA methylation patterns encountered in somatic cells, at regions proximal to CpG islands. Consequently, this leads to variations in gene expression which might affect hPSC usage as organoid generation templates (Doi et al., 2009; Kim et al., 2010; Polo et al., 2010; Bar-Nur et al., 2011; Puri and Nagy, 2012).

Another limitation related to hPSC-derived organoids is that most hPSC cultures are feeder cell-dependent, adding to the complexity of the culturing protocols and increasing the risk of underlying cell culture infections. A shift to feeder-free culturing conditions could increase reproducibility across cell lines and laboratories (Lancaster et al., 2017; Yoon et al., 2019). Due to the above limitations, hPSC-derived organoid cultures need to be constantly compared to independent batches of multiple hPSC lines and adequately assessed for their capacity to produce consistent results, before being put forward as powerful disease model systems.

### Future Perspectives of the Organoid Technology

Since 2009, a 3D *in vitro* culture system for several organs, such as small intestine, colon, stomach, prostate, liver, pancreas, breast, lung, and skin has been established (Sato et al., 2009; Barker et al., 2010; Sato et al., 2011; Karthaus et al., 2014; Boj et al., 2015; Huch et al., 2015; Sachs et al., 2018; Wiener et al., 2018; Sachs et al., 2019), based on stimulating the self-renewal capacity of the underlying stem cell populations. Culturing of the above tissues in defined conditioned media results in the formation of 3D mini-tissues, also called organoids. Those primary tissue-derived organoids can be established from mouse and human tissue of any age, they do not require additional cell types to stimulate growth, are genetically stable and can retain the *in vivo* organization and development of the tissue they derive from. More importantly, they do not depend on iPSC technology and their long-term culture has been optimized through various protocols depending on the tissue (**Figure 1C**) (Rossi et al., 2018).

Patient-derived organoids offer a unique model system, as it resembles the *in vivo* situation more closely than any other cell culture so far. All attempts, however, to generate organoids immediately derived from primary material have been focused on epithelial tissue. Given that the study of neurodegenerative disorders requires the establishment and maintenance of non-epithelial cell cultures, one of the most important future challenges is to adapt the current patient-derived organoid technology to model diseases encountered in non-epithelial tissues. Taking into account the numerous advantages of patient-derived organoids, the field is soon expected to expand this cutting edge technology to encompass non-epithelial tissue. In doing so, the biggest challenge would be to define the optimal media composition supporting the *in vitro* generation and maintenance of patient-derived brain organoids. The next step following the establishment of hPSC-free brain organoids would be to implement means of genetic manipulation and drug delivery, allowing for personalized treatment approaches. Along those lines, and considering the advantages of patient-derived brain organoids with regards to functionality and biosafety, the potential of utilizing the system in regenerative medicine would be greater than any other system so far.

## Discussion

Several attempts have been made to model and pharmacologically target neurodegenerative diseases, such as AD, with the use of brain organoids. So far, brain organoid generation attempts have been mostly focused on somatic cell reprogramming, a process in which patient-derived somatic cells are induced to become hPSCs (Amin and Pasca, 2018). HPSCs can be subsequently differentiated into monolayer neuronal cultures or brain organoids, which are 3D neural cell aggregates resembling various brain regions. In the case of AD, there have been several attempts to generate brain organoids using the hPSC technology (Raja et al., 2016; Pavoni et al., 2018; Gerakis and Hetz, 2019; Qian et al., 2019) and a lot of progress has been made both in modeling the disease and assessing the effectiveness of drugs like γ-secretase inhibitors to reverse AD-related phenotypes. With regards to their differentiation pattern, unguided brain organoids have shown suitability in modeling cell-lineage diversity in whole brain development, whereas directed brain organoids may be fused to form assembloids in order to capture and study processes linked to specific brain regions, including the hippocampal loss in AD (Bagley et al., 2017; Birey et al., 2017; Xiang et al., 2017).

HPSC-derived organoids are accompanied by a series of limitations, such as lack of or limited integration of important cell types (e.g. microglial cells and oligodendrocytes), lack of distinct cortical neuronal layer formation, no evidence of gyrification, nor complex neuronal circuitry (Gerakis and Hetz, 2019; Qian et al., 2019). Additionally, the iPSC technology itself poses limitations with regards to safety, genomic stability, and reproducibility.

Current organoid models are majorly derived from the epithelium of various organs. Established protocols for generating primary tissue-derived organoids could overcome the aging-related issues of hPSC-derived organoids, as primary tissue-derived organoids can be established from mammalian tissue of any age. Additionally, primary tissue-derived organoids are based on more stringent differentiation protocols, in contrast to protocols relying on hPSC self-organization. It has been widely reported that stochasticity in the hPSC differentiation process culminates in unpredictable outcomes in brain organoid cultures, adding to reproducibility issues. The challenge of adapting epithelial organoid generation protocols to meet the requirements of non-epithelial tissue culture still remains.

## Author Contributions

AP conceived the topic and wrote the manuscript. MT, NF, and AAP contributed to writing the manuscript and critically reviewed it.

## Funding

This research is co-financed by Greece and the European Union (European Social Fund- ESF) through the Operational Programme «Human Resources Development, Education and Lifelong Learning» in the context of the project “Reinforcement of Postdoctoral Researchers - 2nd Cycle” (MIS-5033021), implemented by the State Scholarships Foundation (ΙΚΥ).

## Conflict of Interest

The authors declare that the research was conducted in the absence of any commercial or financial relationships that could be construed as a potential conflict of interest.

## References

[B2] Amin N. D. Pasca S. P. (2018). Building Models of Brain Disorders with Three-Dimensional Organoids. Neuron 100 (2), 389–405. 10.1016/j.neuron.2018.10.007 30359604

[B13] Andorfer C. Kress Y. Espinoza M. de Silva R. Tucker K. L. Barde Y. A. (2003). Hyperphosphorylation and aggregation of tau in mice expressing normal human tau isoforms. J. Neurochem. 86 (3), 582–590. 10.1046/j.1471-4159.2003.01879.x 12859672

[B48] Bagley J. A. Reumann D. Bian S. Levi-Strauss J. Knoblich J. A. (2017). Fused cerebral organoids model interactions between brain regions. Nat. Methods 14 (7), 743–751. 10.1038/nmeth.4304 28504681 PMC5540177

[B74] Barker N. Huch M. Kujala P. van de Wetering M. Snippert H. J. van Es J. H. (2010). Lgr5(+ve) stem cells drive self-renewal in the stomach and build long-lived gastric units in vitro. Cell Stem. Cell 6 (1), 25–36. 10.1016/j.stem.2009.11.013 20085740

[B68] Bar-Nur O. Russ H. A. Efrat S. Benvenisty N. (2011). Epigenetic memory and preferential lineage-specific differentiation in induced pluripotent stem cells derived from human pancreatic islet beta cells. Cell Stem. Cell 9 (1), 17–23. 10.1016/j.stem.2011.06.007 21726830

[B10] Bekris L. M. Yu C. E. Bird T. D. Tsuang D. W. (2010). Genetics of Alzheimer disease. J. Geriatr. Psychiatry Neurol. 23 (4), 213–227. 10.1177/0891988710383571 21045163 PMC3044597

[B47] Birey F. Andersen J. Makinson C. D. Islam S. Wei W. Huber N. (2017). Assembly of functionally integrated human forebrain spheroids. Nature 545 (7652), 54–59. 10.1038/nature22330 28445465 PMC5805137

[B75] Boj S. F. Hwang C. I. Baker L. A. Chio I. I. Engle D. D. Corbo V. (2015). Organoid models of human and mouse ductal pancreatic cancer. Cell 160 (1-2), 324–338. 10.1016/j.cell.2014.12.021 25557080 PMC4334572

[B38] Camp J. G. Badsha F. Florio M. Kanton S. Gerber T. Wilsch-Brauninger M. (2015). Human cerebral organoids recapitulate gene expression programs of fetal neocortex development. Proc. Natl. Acad. Sci. U. S. A. 112 (51), 15672–15677. 10.1073/pnas.1520760112 26644564 PMC4697386

[B31] Choi S. H. Kim Y. H. Hebisch M. Sliwinski C. Lee S. D'Avanzo C. (2014). A three-dimensional human neural cell culture model of Alzheimer’s disease. Nature 515 (7526), 274–278. 10.1038/nature13800 25307057 PMC4366007

[B1] Collaborators, G.B.D.D (2019). Global, regional, and national burden of Alzheimer’s disease and other dementias, 1990-2016: a systematic analysis for the Global Burden of Disease Study 2016. Lancet Neurol. 18 (1), 88–106. 10.1016/S1474-4422(18)30403-4 30497964 PMC6291454

[B43] Danjo T. Eiraku M. Muguruma K. Watanabe K. Kawada M. Yanagawa Y. (2011). Subregional specification of embryonic stem cell-derived ventral telencephalic tissues by timed and combinatory treatment with extrinsic signals. J. Neurosci. 31 (5), 1919–1933. 10.1523/JNEUROSCI.5128-10.2011 21289201 PMC6623725

[B29] Dehaene-Lambertz G. Spelke E. S. (2015). The Infancy of the Human Brain. Neuron 88 (1), 93–109. 10.1016/j.neuron.2015.09.026 26447575

[B37] Di Lullo E. Kriegstein A. R. (2017). The use of brain organoids to investigate neural development and disease. Nat. Rev. Neurosci. 18 (10), 573–584. 10.1038/nrn.2017.107 28878372 PMC5667942

[B69] Doi A. Park I. H. Wen B. Murakami P. Aryee M. J. Irizarry R. (2009). Differential methylation of tissue- and cancer-specific CpG island shores distinguishes human induced pluripotent stem cells, embryonic stem cells and fibroblasts. Nat. Genet. 41 (12), 1350–1353. 10.1038/ng.471 19881528 PMC2958040

[B5] Dos Santos Picanco L. C. Ozela P. F. de Fatima de Brito Brito M. Pinheiro A. A. Padilha E. C. Braga F. S. (2018). Alzheimer’s Disease: A Review from the Pathophysiology to Diagnosis, New Perspectives for Pharmacological Treatment. Curr. Med. Chem. 25 (26), 3141–3159. 10.2174/0929867323666161213101126 30191777

[B44] Eiraku M. Watanabe K. Matsuo-Takasaki M. Kawada M. Yonemura S. Matsumura M. (2008). Self-organized formation of polarized cortical tissues from ESCs and its active manipulation by extrinsic signals. Cell Stem Cell 3 (5), 519–532. 10.1016/j.stem.2008.09.002 18983967

[B26] Freude K. Pires C. Hyttel P. Hall V. J. (2014). Induced Pluripotent Stem Cells Derived from Alzheimer’s Disease Patients: The Promise, the Hope and the Path Ahead. J. Clin. Med. 3 (4), 1402–1436. 10.3390/jcm3041402 26237610 PMC4470192

[B14] Gerakis Y. Hetz C. (2019). Brain organoids: a next step for humanized Alzheimer’s disease models?. Mol. Psychiatry 24 (4), 474–478. 10.1038/s41380-018-0343-7 30617271

[B63] Ginhoux F. Prinz M. (2015). Origin of microglia: current concepts and past controversies. Cold Spring Harb. Perspect. Biol. 7 (8), a020537. 10.1101/cshperspect.a020537 26134003 PMC4526747

[B51] Gonzalez C. Armijo E. Bravo-Alegria J. Becerra-Calixto A. Mays C. E. Soto C. (2018). Modeling amyloid beta and tau pathology in human cerebral organoids. Mol. Psychiatry 23 (12), 2363–2374. 10.1038/s41380-018-0229-8 30171212 PMC6594704

[B24] Harasta A. E. Ittner L. M. (2017). Alzheimer’s Disease: Insights from Genetic Mouse Models and Current Advances in Human IPSC-Derived Neurons. Adv. Neurobiol. 15, 3–29. 10.1007/978-3-319-57193-5_1 28674976

[B8] Heppner F. L. Ransohoff R. M. Becher B. (2015). Immune attack: the role of inflammation in Alzheimer disease. Nat. Rev. Neurosci. 16 (6), 358–372. 10.1038/nrn3880 25991443

[B4] Holtzman D. M. Morris J. C. Goate A. M. (2011). Alzheimer’s disease: the challenge of the second century. Sci. Transl. Med. 3 (77), 77sr1. 10.1126/scitranslmed.3002369 21471435 PMC3130546

[B22] Huang Y. A. Zhou B. Wernig M. Sudhof T. C. (2017). ApoE2, ApoE3, and ApoE4 Differentially Stimulate APP Transcription and Abeta Secretion. Cell 168 (3), 427–441 e21. 10.1016/j.cell.2016.12.044 28111074 PMC5310835

[B76] Huch M. Gehart H. van Boxtel R. Hamer K. Blokzijl F. Verstegen M. M. (2015). Long-term culture of genome-stable bipotent stem cells from adult human liver. Cell 160 (1-2), 299–312. 10.1016/j.cell.2014.11.050 25533785 PMC4313365

[B57] Huch M. Knoblich J. A. Lutolf M. P. Martinez-Arias A. (2017). The hope and the hype of organoid research. Development 144 (6), 938–941. 10.1242/dev.150201 28292837

[B65] Hussein S. M. Batada N. N. Vuoristo S. Ching R. W. Autio R. Narva E. Ng S. (2011). Copy number variation and selection during reprogramming to pluripotency. Nature 471 (7336), 58–62. 10.1038/nature09871 21368824

[B28] Israel M. A. Yuan S. H. Bardy C. Reyna S. M. Mu Y. Herrera C. (2012). Probing sporadic and familial Alzheimer’s disease using induced pluripotent stem cells. Nature 482 (7384), 216–220. 10.1038/nature10821 22278060 PMC3338985

[B25] Jorfi M. D'Avanzo C. Tanzi R. E. Kim D. Y. Irimia D. (2018). Human Neurospheroid Arrays for In Vitro Studies of Alzheimer’s Disease. Sci. Rep. 8 (1), 2450. 10.1038/s41598-018-20436-8 29402979 PMC5799361

[B60] Kadoshima T. Sakaguchi H. Nakano T. Soen M. Ando S. Eiraku M. (2013). Self-organization of axial polarity, inside-out layer pattern, and species-specific progenitor dynamics in human ES cell-derived neocortex. Proc. Natl. Acad. Sci. U. S. A. 110 (50), 20284–20289. 10.1073/pnas.1315710110 24277810 PMC3864329

[B54] Kametani F. Hasegawa M. (2018). Reconsideration of Amyloid Hypothesis and Tau Hypothesis in Alzheimer’s Disease. Front. Neurosci. 12, 25. 10.3389/fnins.2018.00025 29440986 PMC5797629

[B77] Karthaus W. R. Iaquinta P. J. Drost J. Gracanin A. van Boxtel R. Wongvipat J. (2014). Identification of multipotent luminal progenitor cells in human prostate organoid cultures. Cell 159 (1), 163–175. 10.1016/j.cell.2014.08.017 25201529 PMC4772677

[B70] Kim K. Doi A. Wen B. Ng K. Zhao R. Cahan P. (2010). Epigenetic memory in induced pluripotent stem cells. Nature 467 (7313), 285–290. 10.1038/nature09342 20644535 PMC3150836

[B50] Kim H. Park H. J. Choi H. Chang Y. Park H. Shin J. (2019). Modeling G2019S-LRRK2 Sporadic Parkinson’s Disease in 3D Midbrain Organoids. Stem Cell Rep. 12 (3), 518–531. 10.1016/j.stemcr.2019.01.020 PMC6410341 30799274

[B15] Kitazawa M. Medeiros R. Laferla F. M. (2012). Transgenic mouse models of Alzheimer disease: developing a better model as a tool for therapeutic interventions. Curr. Pharm. Des. 18 (8), 1131–1147. 10.2174/138161212799315786 22288400 PMC4437619

[B23] Koch P. Tamboli I. Y. Mertens J. Wunderlich P. Ladewig J. Stuber K. (2012). Presenilin-1 L166P mutant human pluripotent stem cell-derived neurons exhibit partial loss of gamma-secretase activity in endogenous amyloid-beta generation. Am. J. Pathol. 180 (6), 2404–2416. 10.1016/j.ajpath.2012.02.012 22510327

[B27] Kondo T. Asai M. Tsukita K. Kutoku Y. Ohsawa Y. Sunada Y. (2013). Modeling Alzheimer’s disease with iPSCs reveals stress phenotypes associated with intracellular Abeta and differential drug responsiveness. Cell Stem. Cell 12 (4), 487–496. 10.1016/j.stem.2013.01.009 23434393

[B36] Lancaster M. A. Renner M. Martin C. A. Wenzel D. Bicknell L. S. Hurles M. E. (2013). Cerebral organoids model human brain development and microcephaly. Nature 501 (7467), 373–379. 10.1038/nature12517 23995685 PMC3817409

[B61] Lancaster M. A. Corsini N. S. Wolfinger S. Gustafson E. H. Phillips A. W. Burkard T. R. (2017). Guided self-organization and cortical plate formation in human brain organoids. Nat. Biotechnol. 35 (7), 659–666. 10.1038/nbt.3906 28562594 PMC5824977

[B66] Laurent L. C. Ulitsky I. Slavin I. Tran H. Schork A. Morey R. (2011). Dynamic changes in the copy number of pluripotency and cell proliferation genes in human ESCs and iPSCs during reprogramming and time in culture. Cell Stem. Cell 8 (1), 106–118. 10.1016/j.stem.2010.12.003 21211785 PMC3043464

[B64] Li Q. Barres B. A. (2018). Microglia and macrophages in brain homeostasis and disease. Nat. Rev. Immunol. 18 (4), 225–242. 10.1038/nri.2017.125 29151590

[B56] Lopez-Otin C. Blasco M. A. Partridge L. Serrano M. Kroemer G. (2013). The hallmarks of aging. Cell 153 (6), 1194–1217. 10.1016/j.cell.2013.05.039 23746838 PMC3836174

[B34] Mansour A. A. Goncalves J. T. Bloyd C. W. Li H. Fernandes S. Quang D. (2018). An in vivo model of functional and vascularized human brain organoids. Nat. Biotechnol. 36 (5), 432–441. 10.1038/nbt.4127 29658944 PMC6331203

[B40] Mariani J. Coppola G. Zhang P. Abyzov A. Provini L. Tomasini L. (2015). FOXG1-Dependent Dysregulation of GABA/Glutamate Neuron Differentiation in Autism Spectrum Disorders. Cell 162 (2), 375–390. 10.1016/j.cell.2015.06.034 26186191 PMC4519016

[B67] Mayshar Y. Ben-David U. Lavon N. Biancotti J. C. Yakir B. Clark A. T. (2010). Identification and classification of chromosomal aberrations in human induced pluripotent stem cells. Cell Stem. Cell 7 (4), 521–531. 10.1016/j.stem.2010.07.017 20887957

[B32] Muguruma K. Nishiyama A. Kawakami H. Hashimoto K. Sasai Y. (2015). Self-organization of polarized cerebellar tissue in 3D culture of human pluripotent stem cells. Cell Rep. 10 (4), 537–550. 10.1016/j.celrep.2014.12.051 25640179

[B21] Muratore C. R. Rice H. C. Srikanth P. Callahan D. G. Shin T. Benjamin L. N. (2014). The familial Alzheimer’s disease APPV717I mutation alters APP processing and Tau expression in iPSC-derived neurons. Hum. Mol. Genet. 23 (13), 3523–3536. 10.1093/hmg/ddu064 24524897 PMC4049307

[B33] Nakano T. Ando S. Takata N. Kawada M. Muguruma K. Sekiguchi K. (2012). Self-formation of optic cups and storable stratified neural retina from human ESCs. Cell Stem. Cell 10 (6), 771–785. 10.1016/j.stem.2012.05.009 22704518

[B12] Nisbet R. M. Polanco J. C. Ittner L. M. Gotz J. (2015). Tau aggregation and its interplay with amyloid-beta. Acta Neuropathol. 129 (2), 207–220. 10.1007/s00401-014-1371-2 25492702 PMC4305093

[B6] Palmer A. M. (2011). Neuroprotective therapeutics for Alzheimer’s disease: progress and prospects. Trends Pharmacol. Sci. 32 (3), 141–147. 10.1016/j.tips.2010.12.007 21256602

[B52] Park J. Wetzel I. Marriott I. Dreau D. D'Avanzo C. Kim D. Y. (2018). A 3D human triculture system modeling neurodegeneration and neuroinflammation in Alzheimer’s disease. Nat. Neurosci. 21 (7), 941–951. 10.1038/s41593-018-0175-4 29950669 PMC6800152

[B41] Pasca S. P. Portmann T. Voineagu I. Yazawa M. Shcheglovitov A. Pasca A. M. (2011). Using iPSC-derived neurons to uncover cellular phenotypes associated with Timothy syndrome. Nat. Med. 17 (12), 1657–1662. 10.1038/nm.2576 22120178 PMC3517299

[B19] Pavoni S. Jarray R. Nassor F. Guyot A. C. Cottin S. Rontard J. (2018). Small-molecule induction of Abeta-42 peptide production in human cerebral organoids to model Alzheimer’s disease associated phenotypes. PloS One 13 (12), e0209150. 10.1371/journal.pone.0209150 30557391 PMC6296660

[B35] Pham M. T. Pollock K. M. Rose M. D. Cary W. A. Stewart H. R. Zhou P. (2018). Generation of human vascularized brain organoids. Neuroreport 29 (7), 588–593. 10.1097/WNR.0000000000001014 29570159 PMC6476536

[B71] Polo J. M. Liu S. Figueroa M. E. Kulalert W. Eminli S. Tan K. Y. (2010). Cell type of origin influences the molecular and functional properties of mouse induced pluripotent stem cells. Nat. Biotechnol. 28 (8), 848–855. 10.1038/nbt.1667 20644536 PMC3148605

[B9] Prokop S. Miller K. R. Heppner F. L. (2013). Microglia actions in Alzheimer’s disease. Acta Neuropathol. 126 (4), 461–477. 10.1007/s00401-013-1182-x 24224195

[B72] Puri M. C. Nagy A. (2012). Concise review: Embryonic stem cells versus induced pluripotent stem cells: the game is on. Stem Cells 30 (1), 10–14. 10.1002/stem.788 22102565

[B42] Qian X. Nguyen H. N. Song M. M. Hadiono C. Ogden S. C. Hammack C. (2016). Brain-Region-Specific Organoids Using Mini-bioreactors for Modeling ZIKV Exposure. Cell 165 (5), 1238–1254. 10.1016/j.cell.2016.04.032 27118425 PMC4900885

[B84] Qian X. Song H. Ming G. L. (2019). Brain organoids: advances, applications and challenges. Development 146 (8). 10.1242/dev.166074 PMC6503989 30992274

[B39] Quadrato G. Nguyen T. Macosko E. Z. Sherwood J. L. Min Yang S. Berger D. R. (2017). Cell diversity and network dynamics in photosensitive human brain organoids. Nature 545 (7652), 48–53. 10.1038/nature22047 28445462 PMC5659341

[B53] Raja W. K. Mungenast A. E. Lin Y. T. Ko T. Abdurrob F. Seo J. (2016). Self-Organizing 3D Human Neural Tissue Derived from Induced Pluripotent Stem Cells Recapitulate Alzheimer’s Disease Phenotypes. PloS One 11 (9), e0161969. 10.1371/journal.pone.0161969 27622770 PMC5021368

[B59] Rambani K. Vukasinovic J. Glezer A. Potter S. M. (2009). Culturing thick brain slices: an interstitial 3D microperfusion system for enhanced viability. J. Neurosci. Methods 180 (2), 243–254. 10.1016/j.jneumeth.2009.03.016 19443039 PMC2742628

[B83] Rossi G. Manfrin A. Lutolf M. P. (2018). Progress and potential in organoid research. Nat. Rev. Genet. 19 (11), 671–687. 10.1038/s41576-018-0051-9 30228295

[B18] Rowe R. G. Daley G. Q. (2019). Induced pluripotent stem cells in disease modelling and drug discovery. Nat. Rev. Genet. 20 (7), 377–388. 10.1038/s41576-019-0100-z 30737492 PMC6584039

[B80] Sachs N. de Ligt J. Kopper O. Gogola E. Bounova G. Weeber F. (2018). A Living Biobank of Breast Cancer Organoids Captures Disease Heterogeneity. Cell 172 (1-2), 373–386 e10. 10.1016/j.cell.2017.11.010 29224780

[B81] Sachs N. Papaspyropoulos A. Zomer-van Ommen D. D. Heo I. Bottinger L. Klay D. (2019). Long-term expanding human airway organoids for disease modeling. EMBO J. 38, e100300. 10.15252/embj.2018100300 PMC6376275 30643021

[B45] Sakaguchi H. Kadoshima T. Soen M. Narii N. Ishida Y. Ohgushi M. (2015). Generation of functional hippocampal neurons from self-organizing human embryonic stem cell-derived dorsomedial telencephalic tissue. Nat. Commun. 6, 8896. 10.1038/ncomms9896 26573335 PMC4660208

[B16] Sasaguri H. Nilsson P. Hashimoto S. Nagata K. Saito T. De Strooper B. (2017). APP mouse models for Alzheimer’s disease preclinical studies. EMBO J. 36 (17), 2473–2487. 10.15252/embj.201797397 28768718 PMC5579350

[B79] Sato T. Vries R. G. Snippert H. J. van de Wetering M. Barker N. Stange D. E. (2009). Single Lgr5 stem cells build crypt-villus structures in vitro without a mesenchymal niche. Nature 459 (7244), 262–265. 10.1038/nature07935 19329995

[B78] Sato T. Stange D. E. Ferrante M. Vries R. G. Van Es J. H. Van den Brink S. (2011). Long-term expansion of epithelial organoids from human colon, adenoma, adenocarcinoma, and Barrett’s epithelium. Gastroenterology 141 (5), 1762–1772. 10.1053/j.gastro.2011.07.050 21889923

[B30] Silbereis J. C. Pochareddy S. Zhu Y. Li M. Sestan N. (2016). The Cellular and Molecular Landscapes of the Developing Human Central Nervous System. Neuron 89 (2), 248–268. 10.1016/j.neuron.2015.12.008 26796689 PMC4959909

[B46] Sloan S. A. Darmanis S. Huber N. Khan T. A. Birey F. Caneda C. (2017). Human Astrocyte Maturation Captured in 3D Cerebral Cortical Spheroids Derived from Pluripotent Stem Cells. Neuron 95 (4), 779–790 e6. 10.1016/j.neuron.2017.07.035 28817799 PMC5890820

[B55] Takahashi M. Miyata H. Kametani F. Nonaka T. Akiyama H. Hisanaga S. (2015). Extracellular association of APP and tau fibrils induces intracellular aggregate formation of tau. Acta Neuropathol. 129 (6), 895–907. 10.1007/s00401-015-1415-2 25869641 PMC4436700

[B58] Takebe T. Enomura M. Yoshizawa E. Kimura M. Koike H. Ueno Y. (2015). Vascularized and Complex Organ Buds from Diverse Tissues via Mesenchymal Cell-Driven Condensation. Cell Stem Cell 16 (5), 556–565. 10.1016/j.stem.2015.03.004 25891906

[B17] Tiscornia G. Vivas E. L. Izpisua Belmonte J. C. (2011). Diseases in a dish: modeling human genetic disorders using induced pluripotent cells. Nat. Med. 17 (12), 1570–1576. 10.1038/nm.2504 22146428

[B11] van Horssen J. Wilhelmus M. M. Heljasvaara R. Pihlajaniemi T. Wesseling P. de Waal R. M. (2002). Collagen XVIII: a novel heparan sulfate proteoglycan associated with vascular amyloid depositions and senile plaques in Alzheimer’s disease brains. Brain Pathol. 12 (4), 456–462. 10.1111/j.1750-3639.2002.tb00462.x 12408231 PMC8095772

[B20] Vazin T. Ball K. A. Lu H. Park H. Ataeijannati Y. Head-Gordon T. (2014). Efficient derivation of cortical glutamatergic neurons from human pluripotent stem cells: a model system to study neurotoxicity in Alzheimer’s disease. Neurobiol. Dis. 62, 62–72. 10.1016/j.nbd.2013.09.005 24055772 PMC4122237

[B3] Vigo D. Thornicroft G. Atun R. (2016). Estimating the true global burden of mental illness. Lancet Psychiatry 3 (2), 171–178. 10.1016/S2215-0366(15)00505-2 26851330

[B82] Wiener D. J. Basak O. Asra P. Boonekamp K. E. Kretzschmar K. Papaspyropoulos A. (2018). Establishment and characterization of a canine keratinocyte organoid culture system. Vet. Dermatol. 29 (5), 375–e126. 10.1111/vde.12541 29963730

[B49] Xiang Y. Tanaka Y. Patterson B. Kang Y. J. Govindaiah G. Roselaar N. (2017). Fusion of Regionally Specified hPSC-Derived Organoids Models Human Brain Development and Interneuron Migration. Cell Stem Cell 21 (3), 383–398 e7. 10.1016/j.stem.2017.07.007 28757360 PMC5720381

[B62] Yakoub A. M. (2019). Cerebral organoids exhibit mature neurons and astrocytes and recapitulate electrophysiological activity of the human brain. Neural Regen. Res. 14 (5), 757–761. 10.4103/1673-5374.249283 30688257 PMC6375034

[B7] Yan Y. Bejoy J. Marzano M. Li Y. (2019). The Use of Pluripotent Stem Cell-Derived Organoids to Study Extracellular Matrix Development during Neural Degeneration. Cells 8 (3), 242. 10.3390/cells8030242 PMC6468789 30875781

[B73] Yoon S. J. Elahi L. S. Pasca A. M. Marton R. M. Gordon A. Revah O. (2019). Reliability of human cortical organoid generation. Nat. Methods 16 (1), 75–78. 10.1038/s41592-018-0255-0 30573846 PMC6677388

